# Sex Differences in the Ergogenic Response of Acute Caffeine Intake on Muscular Strength, Power and Endurance Performance in Resistance-Trained Individuals: A Randomized Controlled Trial

**DOI:** 10.3390/nu16111760

**Published:** 2024-06-04

**Authors:** Juan Jesús Montalvo-Alonso, Carmen Ferragut, Marta del Val-Manzano, David Valadés, Justin Roberts, Alberto Pérez-López

**Affiliations:** 1 Universidad de Alcalá, Facultad de Medicina y Ciencias de la Salud, Departamento de Ciencias Biomédicas, Área de Educación Física y Deportiva, 28801 Madrid, Spain; jesus.montalvo@uah.es (J.J.M.-A.); carmen.ferragut@uah.es (C.F.); marta.val@edu.uah.es (M.d.V.-M.); david.valades@uah.es (D.V.); 2 Cambridge Centre for Sport and Exercise Sciences, School of Psychology and Sport Science, Anglia Ruskin University, Cambridge CB1 1PT, UK; justin.roberts@aru.ac.uk

**Keywords:** sex differences, ergogenic substances, sport nutrition, sport performance, force-velocity curve

## Abstract

Background: This study assessed the impact of acute caffeine intake on muscular strength, power, and endurance performance between resistance-trained male and female individuals according to load in upper- and lower-body exercises. Methods: Here, 76 resistance-trained individuals (38 females, 38 males) participated in a study comparing caffeine and a placebo. Each received either 3 mg/kg of caffeine or a placebo 60 min before tests measuring muscular strength and power through bench press and back squat exercises at different intensities (25%, 50%, 75%, 90% 1RM). Muscular endurance at 65% 1RM was also assessed by performing reps until reaching task failure. Results: Compared to placebo, caffeine increased mean, peak and time to reach peak velocity and power output (*p* < 0.01, η_p_^2^ = 0.242–0.293) in the muscular strength/power test in males and females. This effect was particularly observed in the back squat exercise at 50%, 75% and 90% 1RM (2.5–8.5%, *p* < 0.05, g = 1.0–2.4). For muscular endurance, caffeine increased the number of repetitions, mean velocity and power output (*p* < 0.001, η_p_^2^ = 0.177–0.255) in both sexes and exercises (3.0–8.9%, *p* < 0.05, g = 0.15–0.33). Conclusions: Acute caffeine intake resulted in a similar ergogenic effect on muscular strength, power, and endurance performance in upper- and lower-body exercises for male and female resistance-trained participants.

## 1. Introduction

In the last three decades, the acute ergogenic effect of caffeine on sports performance has been analyzed in numerous studies, becoming one of the sports supplements with the greatest proven benefits [1]. Scientific evidence shows that acute caffeine intake improves muscle strength, power and endurance in upper- and lower-body exercises [2]. However, most of the evidence to date has involved male participants, with female participants being largely underrepresented [3,4]. Additionally, the sex differences in the acute effect of caffeine on muscular strength and power performance have been scarcely explored [4]. Therefore, from the available literature, it is unclear if the ergogenic effects of caffeine translate to female athletes, particularly trained resistance athletes.

Overall, upper- and lower-body strength is greater in males than females by 157% and 60%, respectively, relative to total body mass, in both recreationally active [5] and trained males and females (matched for training status) [6]. Relative to body mass, lower-body strength for female participants is also greater than upper-body strength, a phenomenon absent in males [7]. Despite these differences, in mass-relative terms, males and females increase strength similarly in response to resistance training [8,9]. A neuromuscular overview revealed absolute differences in motor unit (MU) activation between sexes [10,11]. Generally, males exhibit larger type II fibers than females, requiring greater action potential amplitudes to reach a higher threshold and recruit MU, allowing a higher contractile force production than females [12]. Thus, males rely on lower firing rates to modulate contractile force than females [12]. This difference in the neuromuscular profile between males and females may explain the differences observed in the force–velocity curve [13,14], where mean velocity and power are greater in males compared to females at a range of 30% to 70% 1RM [13]. However, when normalizing for strength, no sex differences have been observed in the back squat exercise, at least in terms of power production [14].

Caffeine (1,3,7-trimethylxanthine) is a potent stimulant known to enhance physical performance across a wide range of exercise and sports activities. When consumed at doses of 3 to 6 mg/kg of body mass, caffeine can notably improve muscular strength and power output in individuals trained in resistance exercises [2,15,16,17]. Specifically, it increases mean velocity production (V_mean_) by 2.9% during bench press exercises at 30% of one-repetition maximum (1RM) [15], and by 5.4% to 8.5% in both bench press and back squat exercises at 25% and 50% 1RM, though this effect is not observed at higher intensities like 75% or 90% 1RM [16]. Similarly, improvements are seen at 25%, 50%, and 75% 1RM, but not at 90% 1RM [17]. However, these studies focused on male participants, and when female participants were involved, differences at 75% and 90% 1RM (but not at 25% and 50% 1RM) were found in V_mean_ and mean power production (W_mean_), which may highlight potential sex differences [2]. Furthermore, a meta-analysis by Warren et al. [18] demonstrated that doses of 5 to 6 mg/kg of body mass led to a significant 14% improvement in muscular endurance across 19 out of 23 studies. This ergogenic effect was particularly evident in resistance-trained individuals who underwent bench press and back squat exercises until task failure at 60% 1RM [19] and at 85% 1RM [2], although sex-specific differences were not explored in that study.

There is a consensus that the ergogenic effect of caffeine occurs mainly through a central mechanism by antagonizing adenosine receptors, thereby facilitating increased muscle fiber conducting velocity and motor unit recruitment [20], although these effects may vary across different muscle groups [21,22,23,24], presumably eliciting a greater increase in force production in large compared to small muscle groups. Previous studies have supported this idea in isokinetic tasks [25], and indirectly in isotonic tasks [2]. Furthermore, one cannot avoid a potential peripheral ergogenic effect of caffeine, stimulating the inhibition of phosphodiesterase and calcium ion (Ca^2+^) release from the sarcoplasmic reticulum, increasing sodium–potassium pump activity or antagonizing benzodiazepine receptors in skeletal muscle [26]. However, most of this evidence is extracted from male participants, and the potential sex differences in caffeine ergogenicity and its mechanism of action in males and females remain scarcely studied.

Hence, the purpose of this study was to investigate how acute caffeine consumption affects muscular strength, power, and endurance performance in resistance-trained individuals, specifically focusing on potential differences between males and females across various loads and upper- and lower-body exercises. It was hypothesized that acute caffeine ingestion would improve strength, power and endurance at moderate to high loads, particularly in greater muscle size groups, to a similar extent in both sexes.

## 2. Materials and Methods

### 2.1. Participants

Here, 76 resistance-trained participants (38 females and 38 males) of the same ethnicity (white) who were moderate caffeine consumers (5.6 ± 4.25 mg/kg/day) [27] and had completed a supervised and structured resistance training program (females 3.0 ± 1.7 y, males 2.5 ± 1.3 y), including bench press (1RM/kg females 1.09 ± 0.11 and males 1.18 ± 0.20) and back squat exercise (1RM/kg females 1.41 ± 0.25 and males 1.59 ± 0.26), were recruited for this investigation using social networks and posters mainly from sport facilities near the laboratory. The inclusion/exclusion criteria for this study were as follows: (a) participants aged between 18 and 35 years; (b) absence of neuromuscular, musculoskeletal, neurological, immunological, or cardio-metabolic disorders; (c) a minimum of 6 months of experience in resistance training, with a training frequency of at least 3 days per week over the past 3 months, as confirmed by a questionnaire; (d) no use of medication, drugs, stimulants, or other sports supplements during the trial. Twenty out of the thirty-eight female participants began the trial during the follicular phase of their menstrual cycle.

Prior to enrollment in the study, participants were thoroughly briefed on all experimental procedures, potential risks, and any associated discomfort. Following this explanation, participants provided written informed consent before proceeding with the study. The study design and protocol adhered to the tenets of the Declaration of Helsinki and was approved by the University Ethical Committee of Investigation.

### 2.2. Experimental Design

A triple-blind, placebo-controlled, crossover, counterbalanced and randomized experimental design was used in this investigation. Participants attended the laboratory on three separate occasions. During the initial visit, participants underwent assessments related to their dietary habits, physical activity, and body composition. Additionally, this visit included a familiarization session where a personal trainer evaluated the participants’ bench press and back squat exercises. The trainer also determined each participant’s one-repetition maximum (1RM) for both exercises. Bench press and back squat exercises were chosen as they target major muscle groups in the upper and lower body, respectively, and have been previously used to investigate the performance-enhancing effects of caffeine on muscular strength, power, and endurance [28]. Data obtained from visit one were not used to test the hypothesis of the study.

During visits two and three, participants took part in two separate trials with a minimum of 72 h between each to ensure full recovery. During visit two, participants were randomly assigned to receive either 3 mg/kg of body mass of caffeine (CAF) or a placebo (PL). Then, during visit three, participants received the opposite supplement from that which they had had in visit two, following a crossover design. The sequence of these trials was randomized for each participant using www.randomized.org to determine the experimental condition order. To maintain blinding throughout the study, an external researcher assigned an alphanumeric code to each sequence. These codes were kept by the external researcher, to blind participants and researchers during the trials and data analysis. The codes were revealed only after the completion of statistical analysis

### 2.3. Experimental Protocol

#### 2.3.1. Body Composition, Dietary and Physical Activity Habits

One week prior to the initial experimental trial, participants attended a familiarization session at the laboratory. During each visit, body composition was assessed using electric bioimpedance (Tanita MC-780MA, Tanita Corporation of America Inc., Arlington Heights, IL, USA). Body mass measurements were used to calculate individualized supplement dosages

Dietary habits were evaluated using a 24-h dietary recall, and physical activity habits were assessed using the International Physical Activity Questionnaire (IPAQ) [29]. Starting 24 h before the familiarization session and continuing until the conclusion of the experimental trial, participants were instructed to abstain from consuming caffeine, stimulants, alcohol, and engaging in strenuous exercise. They were also asked to maintain a consistent sleep schedule and dietary pattern. Sleep patterns were evaluated using a self-report questionnaire, while dietary and fluid intakes were recorded using a 24-h recall questionnaire. The dietary intake from the 24 h preceding each laboratory visit was replicated. Additionally, both the familiarization and experimental trials were conducted at the same time of day to minimize the influence of circadian rhythms on the study outcomes.

#### 2.3.2. Supplementation Protocol

The supplementation protocol began 60 min prior to the trial [30]. Participants consumed either caffeine (CAF) at a dosage of 3 mg/kg of body mass (HSN, Granada, Spain) or a placebo (PLA) consisting of 3 mg/kg of maltodextrin (HSN, Granada, Spain). The supplements were dissolved in 150 mL of tap water and flavored with a calorie-free additive to mask the supplements’ flavor/bitterness and smell (MyProtein, Northwich, UK). The beverages were served in opaque shaker bottles to ensure that participants and researchers were unaware of which supplement was being consumed

#### 2.3.3. One-Repetition Maximum (1RM)

Bench press and back squat exercises’ one-repetition maximum (1RM) were obtained to determine the load (kg) corresponding to 25%, 50%, 75% and 90% 1RM for each participant, performed using a standard Smith machine (Multipower, Technogym, Barcelona, Spain). To determine the loads corresponding to 25%, 50%, 75%, and 90% of their one-repetition maximum (1RM) for bench press and back squat exercises, participants underwent a 1RM test using a standard Smith machine (Multipower, Technogym, Spain). The procedure of the 1RM test began with an initiation load of 20 kg for males and 10 kg for females. This load was incrementally increased by 15–10 kg until the Vmean reached 0.5 m/s for bench press and 0.8 m/s for back squat, as measured by a linear transducer (Encoder, Chronojump Boscosystem, Barcelona, Spain). Smaller increments (≤5 kg) were then used to fine-tune and determine the 1RM. Following a 20-min passive recovery period, participants engaged in a familiarization session where they performed the same tests in the same sequence as in the experimental trials. A pilot study was used to determine the recovery time to ensure that participants were mentally and physically prepared for the subsequent tests. Then, participants underwent a standardized warm-up consisting of 10 min of dynamic stretches and joint mobilization exercises before proceeding to the muscular power, strength, and endurance tests.

#### 2.3.4. Muscular Strength and Power Test

The test consisted of measuring bar velocity displacement using a Smith machine (Multipower, Technogym, Spain), equipped with a linear encoder attached to the bar (Encoder, Chronojump Boscosystem, Italy) [31,32], to assess velocity and power output at four incremental loads corresponding to 25%, 50%, 75%, and 90% of the participants’ one-repetition maximum (1RM) for bench press and back squat exercises. During each trial, participants undertook three attempts at 25% 1RM, two attempts at 50% 1RM, and one attempt each at 75% and 90% 1RM. For each attempt, participants were instructed to control the eccentric phase, pause for 2 s in the isometric phase, and then perform the concentric phase with maximal velocity achievable, maintaining a consistent range of motion for each exercise. A recovery period of three minutes was provided between sets to ensure participants’ readiness for subsequent attempts and to minimize fatigue effects during the testing session. This standardized protocol was followed to accurately evaluate performance across varying loads and exercise intensities.

#### 2.3.5. Muscular Endurance Test

Participants were instructed to perform one set of bench press and back squat exercises at 65% 1RM, aiming to complete as many repetitions as possible until reaching task failure. During each repetition, participants were asked to control the eccentric phase, hold for 2 s in the isometric phase, and then perform the concentric phase with maximal velocity, maintaining a consistent range of motion for each exercise. After completing a set or exercise type, participants rested passively for 5 min before proceeding to the next set or exercise. This recovery period was implemented to minimize fatigue and optimize performance during each exercise session.

#### 2.3.6. Isometric Strength and Vertical Jump

The isometric handgrip and isometric mid-thigh pull tests were performed using a handgrip and back/legs dynamometers (Grip-D, Takei Scientific Instruments Co., Ltd., Tokyo, Japan) [33,34,35]. These isometric tests were selected to measure maximal force production in single- and multi-joint exercises since individuals produce greater force in maximal isometric than in concentric actions. Each test consisted of three repetitions where participants exerted maximal muscular tension for 5 s per attempt, followed by 30 s of passive recovery between repetitions. Additionally, countermovement jump (CMJ) tests were conducted on a force platform (Kistler 9229A, Winterthur, Switzerland). Participants performed three CMJ attempts without using arm swing, with 1 min of passive recovery between attempts. The average and best jump heights achieved by each participant were recorded during these tests

#### 2.3.7. Questionnaires and Scales

Upon completion of the familiarization session and experimental trials, participants were asked to complete a questionnaire regarding their perception of power, endurance, energy, exertion, as well as any discomfort experienced in the heart, muscles, or gastrointestinal system [36]. This questionnaire employed a 1- to 10-point scale for each item, where a rating of 1 indicated the minimal amount and 10 indicated the maximal amount of the respective item. Participants were briefed beforehand on the meaning of each point on the scale. Furthermore, participants’ mood was assessed using a condensed version of the Profile of Mood States questionnaire (POMS) [37,38,39] and the Subjective Vitality Scale (SVS) [37,40]. They were presented with 29 mood-related items and asked to rate their current feelings on a Likert scale ranging from 0 (not at all) to 4 (extremely) in response to the question, “How do you feel at this moment?” This assessment covered six scales: tension, depression, anger, vigor, fatigue, and confusion. Lastly, the questionnaire included a specific query to evaluate the effectiveness of the blinding procedure employed during the study to ensure that participants remained unaware of which supplement they had received.

### 2.4. Statistical Analysis

The sample size calculation determined that 58 participants (29 females and 29 males) was sufficient to achieve the study’s objective, with a desired effect size of 0.3 (α = 0.05; 1 − β = 0.80), calculated using G*Power software (v3.1, Dusseldorf University, Dusseldorf, Germany). In total, 76 participants were recruited and completed the study protocol.

Data analysis was conducted using the statistical software SPSS v29.0 (SPSS Inc., Chicago, IL, USA), and figures were created using GraphPad Prism (v8, GraphPad Software Inc., La Jolla, CA, USA). The normality of the data was assessed using the Shapiro–Wilk test (*p* > 0.05). Muscular strength and power outcomes were analyzed using a four-way repeated measures ANOVA according to supplement (CAF vs. PLA), sex (female vs. male), load (25%, 50%, 75%, and 90% of 1RM), and exercise type (bench press vs. back squat). Muscular endurance was analyzed using a three-way repeated measures ANOVA based on supplement (CAF vs. PLA), sex (female vs. male), and exercise type (bench press vs. back squat). Mauchly’s test of sphericity was applied prior to ANOVA, and if the assumption of sphericity was violated, the degrees of freedom were adjusted using the Huynh–Feldt correction. Post hoc analyses were conducted using the Holm–Bonferroni correction method when significant differences were observed. Furthermore, the McNemar test was utilized to identify differences in side effects experienced after consuming the beverages.

Values are presented as mean ± standard deviation (SD), and statistical significance was set at *p* < 0.05. Effect sizes (ES) were calculated using partial eta squared (η_p_^2^) for the two-way repeated measures ANOVA and Hedges’s g for partial comparisons.

## 3. Results

Table 1 shows differences among experimental conditions regarding body composition, dietary intake habits and physical activity habits. No supplement effects were found in any of the variables analyzed, except in tension, where CAF intake increased this value in females (+27%, *p* < 0.05; η_p_^2^ > 0.152). As expected, sex differences were found in total energy intake (+36% in males vs. females, *p* < 0.001, η_p_^2^ = 0.436), body mass (+23% in male vs. females, *p* < 0.001, η_p_^2^ = 0.634), fat mass (+28% in male vs. females, *p* = 0.004, η_p_^2^ = 0.213) and fat-free mass (+31% in male vs. females, *p* < 0.001, η_p_^2^ = 0.436).

Moreover, 48% (37 of 76) of the participants correctly guessed the order of the trials. Also, 50% (19 of 38) of female participants and 47% (18 of 38) of male participants guessed the supplements ingested, not reporting statistical differences between sexes.

### 3.1. Muscular Strength and Power

**Figure 1:**
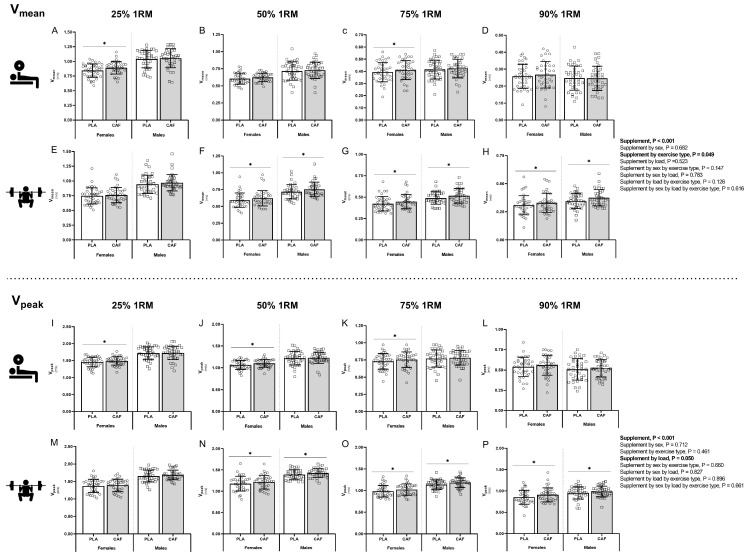
Muscular strength and power test differences in mean and peak velocity (V_mean_ and V_peak_) between CAF and PLA trials according to sex, exercise type and load. V_mean_ performed in the bench press at 25% 1RM (**A**), 50% 1RM (**B**), 75% 1RM (**C**), 90% 1RM (**D**) and the back squat exercise at 25% 1RM (**E**), 50% 1RM (**F**), 75% 1RM (**G**) and 90% 1RM (**H**). V_peak_ performed in the bench press at 25% 1RM (**I**), 50% 1RM (**J**), 75% 1RM (**K**), 90% 1RM (**L**) and the back squat exercise at 25% 1RM (**M**), 50% 1RM (**N**), 75% 1RM (**O**) and 90% 1RM (**P**). * *p* < 0.05 CAF compared to PLA. Abbreviations: CAF, caffeine; PLA, placebo; V_mean_, mean velocity; V_peak_, peak velocity.

Differences in mean and peak velocity (V_mean_ and V_peak_) between CAF and PLA trials according to sex, exercise type and load are illustrated in Figure 1. An overall supplement effect was detected for V_mean_ (*p* < 0.001; η_p_^2^ = 0.261) and V_peak_ (*p* < 0.001; η_p_^2^ = 0.242), although no supplement by sex interaction effect was found for V_mean_ (*p* = 0.682; η_p_^2^ = 0.170) or V_peak_ (*p* = 0.712; η_p_^2^ = 0.137). Nonetheless, for V_mean_, a supplement by exercise type interaction effect (*p* = 0.049; η_p_^2^ = 0.086), and for V_peak_ a supplement by load interaction effect, were found (*p* = 0.050; η_p_^2^ = 0.084). Partial comparison revealed that V_mean_ increased in the back squat exercise in males and females at 50% 1RM (males 4.5%, *p* = 0.001, g = 1.70; females 4.6%, *p* < 0.001, g = 1.87), 75% 1RM (males 5.2%, *p* = 0.001, g = 1.80; females 5.7%, *p* < 0.001, g = 2.04) and 90% 1RM (males 7.9%, *p* = 0.001, g = 2.36; females 7.8%, *p* = 0.030, g = 1.48). However, in the bench press exercise, V_mean_ increased in females at 25% 1RM (5.2%, *p* < 0.001, g = 2.14) and 75% 1RM (3.9%, *p* = 0.027, g = 1.28), an increase that was not statistically significant in males at 25% 1RM (2.5%, *p* > 0.05) and 75% 1RM (2.1%, *p* > 0.05).

In V_peak_, a similar effect pattern was found when partial comparisons were evaluated. V_peak_ increased in the back squat exercise in males and females at 50% 1RM (males 2.5%, *p* = 0.019, g = 1.27; females 2.8%, *p* = 0.005, g = 1.61), 75% 1RM (males 3.9%, *p* = 0.001, g = 2.33; females 3.9%, *p* = 0.002, g = 1.35) and 90% 1RM (males 4.3%, *p* = 0.023, g = 1.69; females 5.8%, *p* = 0.005, g = 2.11).

Moreover, in time to reach V_peak_, supplement (*p* < 0.001; η_p_^2^ = 0.993), supplement by exercise type (*p* < 0.001; η_p_^2^ = 0.296), supplement by load (*p* < 0.001; η_p_^2^ = 0.297) and supplement by exercise type by load (*p* < 0.001; η_p_^2^ = 0.296) interactions were found. This effect was observed in both sexes, but only in the back squat exercise at 50% (males 2.5%, *p* < 0.001, g = 1.87; females 2.9%, *p* = 0.001, g = 1.79), 75% 1RM (males 4.6%, *p* = 0.009, g = 1.58; females 3.8%, *p* = 0.003, g = 1.96) and 90% 1RM (males 2.7%, *p* < 0.001, g = 2.4; females 2.7%, *p* < 0.001, g = 0.94).

**Figure 2:**
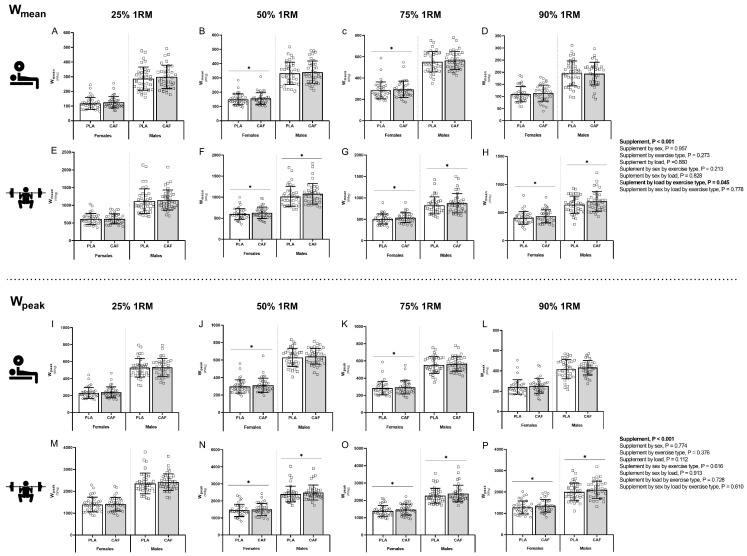
Muscular strength and power tests differences in mean and peak velocity (W_mean_ and W_peak_) between CAF and PLA trials according to sex, exercise type and load. W_mean_ performed in the bench press at 25% 1RM (**A**), 50% 1RM (**B**), 75% 1RM (**C**), 90% 1RM (**D**) and the back squat exercise at 25% 1RM (**E**), 50% 1RM (**F**), 75% 1RM (**G**) and 90% 1RM (**H**). W_peak_ performed in the bench press at 25% 1RM (**I**), 50% 1RM (**J**), 75% 1RM (**K**), 90% 1RM (**L**) and the back squat exercise at 25% 1RM (**M**), 50% 1RM (**N**), 75% 1RM (**O**) and 90% 1RM (**P**). * *p* < 0.05 CAF compared to PLA. Abbreviations: CAF, caffeine; PLA, placebo; W_mean_, mean power output; W_peak_, peak power output.

Differences in mean and peak power (W_mean_ and W_peak_) between CAF and PLA trials according to sex, exercise type and load are illustrated in Figure 2. A supplement effect was detected for W_mean_ (*p* < 0.001; η_p_^2^ = 0.293) and W_peak_ (*p* < 0.001; η_p_^2^ = 0.242), but no supplement by sex effect was found for W_mean_ (*p* = 0.957; η_p_^2^ < 0.01) or W_peak_ (*p* = 0.774; η_p_^2^ = 0.01). However, for W_mean_, a supplement by exercise type by load interaction effect was observed (*p* = 0.045; η_p_^2^ = 0.141). Partial comparison revealed that W_mean_ increased in the bench press exercise in females at 50% 1RM (4.1%, *p* = 0.030, g = 1.07) and 75% 1RM (3.9%, *p* = 0.033, g = 1.00), an increase that was not statistically significant in males at 50% 1RM (2.4%, *p* > 0.05) and 75% 1RM (3.0%, *p* > 0.05). In the back squat exercise, W_mean_ increased in males and females at 50% 1RM (males 5.3%, *p* = 0.002, g = 1.35; females 4.6%, *p* = 0.040, g = 1.27), 75% 1RM (males 5.7%, *p* = 0.001, g = 1.41; females 5.5%, *p* < 0.001, g = 1.53) and 90% 1RM (males 8.5%, *p* = 0.002, g = 2.43; females 6.5%, *p* = 0.028, g = 1.68).

For W_peak_, a similar effect pattern was found when partial comparisons were evaluated. W_peak_ increased in the bench press exercise in females at 50% 1RM (4.4%, *p* < 0.001, g = 1.35) and 75% 1RM (3.2%, *p* = 0.026, g = 1.08), an increase that was not statistically significant in males at 50% 1RM (2.2%, *p* > 0.05) and 75% 1RM (2.1%, *p* > 0.05). In the back squat exercise, W_peak_ increased in males and females at 50%1RM (males 3.5%, *p* = 0.022, g = 1.01; females 3.4%, *p* = 0.012, g = 1.15), 75% 1RM (males 5.1%, *p* = 0.003, g = 1.50; females 5.1%, *p* = 0.003, g = 1.51) and 90% 1RM (males 4.8%, *p* = 0.034, g = 1.52; females 6.0%, *p* = 0.006, g = 1.96).

Moreover, in time to reach W_peak_, supplement (*p* < 0.001; η_p_^2^ = 0.273) and supplement by exercise type effects were identified (*p* = 0.002; η_p_^2^ = 0.132). This effect was observed in both sexes but only in the back squat exercise at 50% (males 6.9%, *p* < 0.001, g = 2.03; females 6.0%, *p* = 0.001, g = 1.96), 75% 1RM (males 5.5%, *p* = 0.008, g = 1.73; females 6.1%, *p* = 0.004, g = 1.88) and 90% 1RM (males 10.3%, *p* = 0.001, g = 2.69; females 7.0%, *p* = 0.029, g = 2.53). No sex differences were found in this variable.

### 3.2. Muscular Endurance

**Figure 3:**
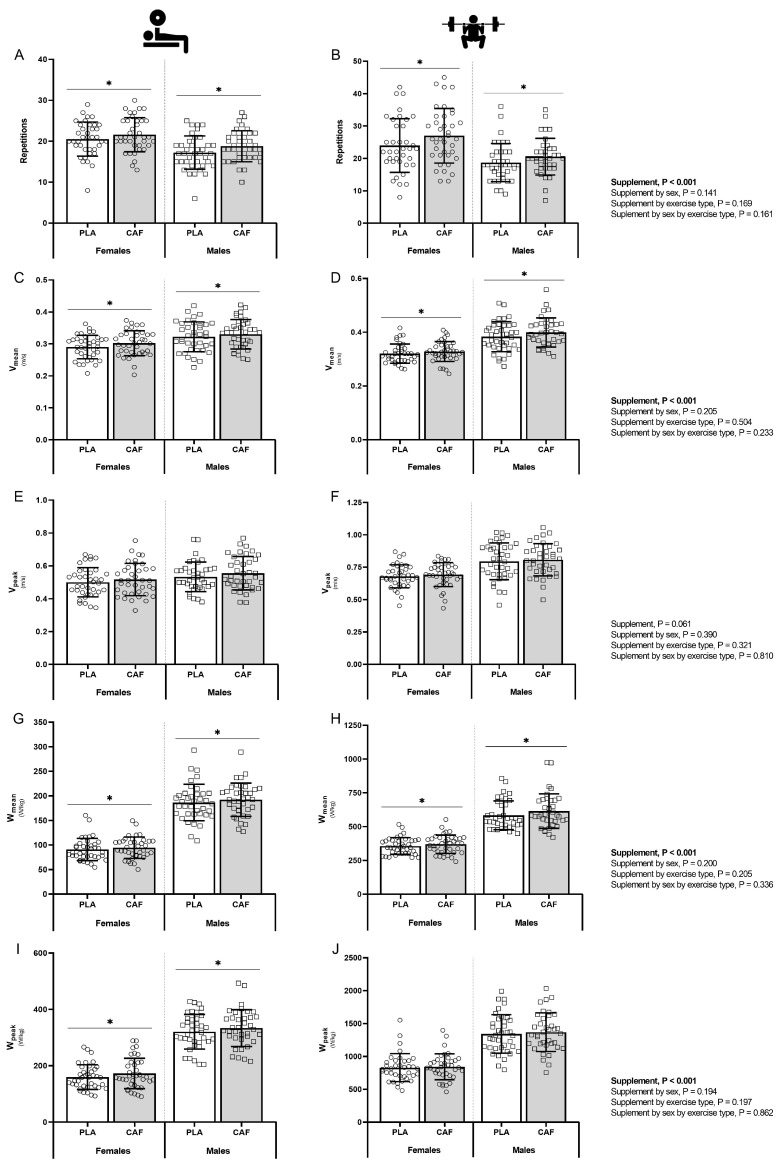
Muscular endurance test differences in the number of repetitions, mean and peak velocity and power output between CAF and PLA trials according to sex and exercise. Number of repetitions performed in bench press (**A**) and back squat exercise (**B**). Mean velocity (V_mean_) performed in the bench press (**C**) and back squat exercise (**D**). Peak velocity output (V_peak_) performed in the bench press (**E**) and back squat exercise (**F**). Mean power output (W_mean_) performed in the bench press (**G**) and back squat exercise (**H**). Peak power output (W_peak_) performed in the bench press (**I**) and back squat exercise (**J**). * *p* < 0.05 CAF compared to PLA. Abbreviations: CAF, caffeine; PLA, placebo; V_mean_, mean velocity; V_peak_, peak velocity; W_mean_, mean power output; W_peak_, peak power output.

Differences in muscular endurance between CAF and PLA trials according to sex, load and exercise type are shown in Figure 3. Supplement effect was detected in the number of repetitions (Reps, *p* < 0.001; η_p_^2^ = 0.177), V_mean_ (*p* < 0.001; η_p_^2^ = 0.255), W_mean_ (*p* < 0.001; η_p_^2^ = 0.198) and W_peak_ (*p* < 0.001; η_p_^2^ = 0.187). However, no supplement by sex effect was found. Partial comparison revealed that reps increased in both sexes for the bench press (males 8.1%, *p* = 0.001, g = 0.325; females 6.0%, *p* = 0.003, g = 0.254) and back squat exercise (males 8.9%, *p* = 0.001, g = 0.310; females 7.9%, *p* = 0.041, g = 0.199). This effect was also observed in V_mean_ for the bench press (males 3.5%, *p* = 0.005, g = 0.251; females 3.0%, *p* = 0.040, g = 0.211) and back squat exercise (males 3.6%, *p* = 0.010, g = 0.184; females 4.0%, *p* = 0.009, g = 0.312) and in W_mean_ for the bench press (males 3.0%, *p* = 0.013, g = 0.154; females 3.8%, *p* = 0.011, g = 0.160) and back squat exercise (males 4.3%, *p* = 0.004, g = 0.181; females 3.1%, *p* = 0.050, g = 0.190). For W_peak_, CAF increased performance compared to PLA for the bench press (males 3.9%, *p* = 0.006, g = 0.199; females 3.4.%, *p* = 0.050, g = 0.111) but not the back squat exercise (males 1.9% and females 1.7%, *p* > 0.05). No statistically significant supplement effect was found in V_peak_, time to reach V_peak_ or time to reach W_peak_.

### 3.3. Isometric Strength and Vertical Jump

A supplement effect was detected in isometric handgrip strength in the dominant hand (*p* = 0.016, η_p_^2^ = 0.159); an effect that was observed in males (3.1%, *p* = 0.027, g = 0.219) but not in female participants (1.6%, *p* = 0.158). No other supplement or supplement by sex effect was found in isometric handgrip strength in the non-dominant hand (*p* = 0.461), the isometric mid-thigh pull test (~1.9%, *p* = 0.117) or the CMJ test (~2.5%, *p* = 0.462).

### 3.4. Questionnaires and Scales

CAF stimulated a statistically significant increase in perceived power in both sexes (3.2 ± 0.7 vs 2.9 ± 0.7; *p* = 0.040; g = 0.312), but no differences were found in endurance (3.2 ± 0.8 vs 3.1 ± 0.7; *p* = 0.020) or fatigue perception compared to placebo (3.2 ± 0.9 vs 3.4 ± 0.9; *p* = 0.089). Moreover, compared to PLA, no statistical differences in side effects were found for CAF in mood state, nervousness, activeness, insomnia, gastrointestinal discomfort, headache or irritability.

## 4. Discussion

The study aimed to investigate the sex differences in the acute effect of CAF on upper- and lower-body muscular strength, power, and endurance performance at different loads in resistance-trained athletes. The key finding of this investigation was that the acute consumption of a low dose of caffeine (3 mg/kg) resulted in improvements in muscular strength, power, and endurance, characterized by increases in mean and peak velocity and power production. Interestingly, these effects were consistent across both sexes and were particularly pronounced during lower-body exercises, specifically the back squat, especially at moderate to high loads (50–90% 1RM).

Previous studies have compared the acute effects of CAF on aerobic and anaerobic performance in both male and female individuals, and reported similar ergogenic effects in both sexes [41,42,43]. However, no study has evaluated the sex differences in the ergogenic effect of CAF on muscular strength, power and endurance performance. Several studies have shown an increase in V_mean_, V_peak_, W_mean_ or W_peak_ in upper- and lower-body strength and power exercises after acute ingestion of 3–6 mg/kg of CAF at a large range of loads from 25% 1RM to 90% 1RM [2,15,16,17]. However, the vast majority of this evidence has been obtained from male participants, with female participants being underrepresented. Nonetheless, some studies have evaluated the ergogenicity of CAF in females (2 to 6 mg/kg of BM) on muscular strength, power or endurance performance [23,44,45,46,47,48]. These studies evaluated muscular strength only at 1RM of leg press in 10 teenage female karate athletes [44], 1RM of bench press in 21 resistance-trained females [46] and 15 young resistance-training females [23,47], 1RM in squat and bench press in 8 young resistance-trained females [48] and 1RM of pull-down, back squat and bench press in 8 resistance-trained females [45]. These studies revealed an ergogenic effect of CAF that was more pronounced in the upper- than the lower-body 1RM test. However, the potential ergogenic effect of CAF on more points of the force–velocity curve has been scarcely studied. Filip-Stachnik et al. [49] observed an increase in the mean velocity of the bench press after 6 mg/kg CAF compared to the control trial but not to the placebo trial. Romero-Moraleda et al. [50] found an ergogenic effect of 3 mg/kg of BM of CAF in half squat exercise at 60%1RM from 1.4 to 5.0% but not at 20, 40, or 80%1RM in 13 trained athletes. Ruiz-Fernández et al. [2] reported a similar ergogenic effect of CAF between male and female resistance-trained participants in muscular strength and power by increasing mean velocity and power output, particularly at high loads (≥75% 1RM), and in lower- compared to upper-body exercises (back squat vs. bench press). However, in this study, sex differences were not reported [2]. In the present study, we found that female resistance-trained athletes’ responses to acute CAF intake were similar in back squat exercises compared to males, improving mean and peak velocity and power production at 50%, 75% and 90% 1RM. This is aligned with previous studies using low doses of CAF; Ruiz-Fernández et al. [2] found an increase in V_mean_ and W_mean_ at 75% and 90% 1RM in the same exercise, and Romero-Moraleda et al. [50] found an ergogenic effect at 60% 1RM, despite the fact that in this study the exercise selected was a half squat. In contrast, when the bench press exercise was analyzed, our study reported that the ergogenic effect of CAF (1.5 to 4.1%) was similar to that in previous studies [2,15,16,17], but less pronounced when compared to the effect caused by this substance in the back squat exercise of this study. Nonetheless, despite no sex differences being found in the bench press exercise, only females reported a statistically significant ergogenic effect of CAF in mean and peak velocity at 25% and 75% 1RM, and in mean and peak power output at 50 and 75% 1RM. Previous studies have reported that females’ responses to resistance training are higher than males in upper-body exercises for untrained populations, exhibiting a higher capacity to increase strength [7]. However, the participants of this study cannot be categorized as untrained since they were involved in a resistance training program for >2 years and reported a relative 1RM to bench press (1RM/kg of body mass) of >1.00. Another potential explanation could be the slight differences in strength between sexes in this exercise (bench press 1RM/kg, females, 1.09 ± 0.11 and males 1.18 ± 0.20), since strength can influence velocity and power production at loads higher than 60% 1RM [14]. Nonetheless, further studies are required to explore the potential differences in muscular strength and power found in female participants in upper-body exercises.

We found a significant supplement by exercise type effect in V_mean_ and a supplement by exercise type and by load effect in W_mean_ in both cases, showing a greater ergogenic effect of CAF in the back squat compared to the bench press exercise. There is a consensus that CAF acts through a central mechanism by antagonizing adenosine receptors, thereby increasing muscle fiber conducting velocity and motor unit recruitment [20]. However, these effects can vary depending on the muscle group involved [21]. Studies evaluating the impact of caffeine on elbow flexor muscles have not shown an ergogenic effect [51], whereas those focusing on knee extensor muscles (such as the quadriceps) have demonstrated performance improvements [22,23]. Notably, muscle activation during maximal voluntary contraction (MVC) tends to be lower in larger muscle groups (like the knee extensors) compared to smaller muscle groups (such as ankle plantar flexors) [24]. Therefore, if caffeine’s ergogenic effect occurs through the CNS, we would expect a greater increase in force production in larger muscle groups due to enhanced motor unit recruitment and muscle fiber conduction velocity. Conversely, if CAF acts directly on muscles, its effect should be similar across different muscle groups [24,25]. Previous research has supported this concept in isokinetic settings [25] and indirectly in dynamic tasks [2]. In this study, we compared the effects of caffeine during back squat (mainly involving the quadriceps) and bench press exercises (primarily engaging the pectorals). Our findings reveal a more pronounced ergogenic effect of low doses of caffeine (3 mg/kg of body mass) on muscular strength and power, indicated by increasing V_mean_ and W_mean_, relative to muscle group size (quadriceps vs. pectorals). Consequently, our results support the hypothesis that CAF enhances force and power production by stimulating the CNS, leading to a greater increase in motor unit recruitment in larger muscle groups compared to smaller muscle groups, with these effects appearing to be consistent regardless of sex.

Our study also evaluated muscular endurance, reporting that CAF increased the number of repetitions, V_mean_, W_mean_ and W_peak_ in both male and female resistance-trained participants to a similar extent. Muscle endurance is a critical quality in resistance exercise involving several sports modalities since it allows for the maintenance of force and power production to a given load for an extended time. According to previous systematic reviews and meta-analyses, CAF has been shown to enhance muscular endurance by approximately 6–7% [52]. This improvement is primarily attributed to an increase in the number of repetitions completed per set following the acute consumption of CAF. However, although no study has compared the ergogenic effect of CAF according to sex, some studies have evaluated the effect of the ergogenicity of CAF in females (2 to 6 mg/kg of BM) on muscular endurance performance [23,44,46,47,48]. Unfortunately, these studies were focused on examining muscular endurance by performing one set at 40% 1RM in 8 young resistance-trained females [48], one set at 50% 1RM in 21 resistance-trained females [46] or one set at 60% in 10 teenage female karate athletes [44] and 15 young resistance-training females [23,47]. However, other evidence expands these effects to velocity and power production at 85% 1RM for bench press [53] and back squat exercises [2]. Our results align with these studies since, compared to PLA, CAF intake was improved in male and female participants muscular endurance at 65% 1RM by increasing the number of repetitions in bench press (8.1% vs. 6.0%) and back squat (8.9 and 7.9%), V_mean_ in bench press (3.5% and 3.0%) and back squat (3.6 vs. 4.0%) and W_mean_ in bench press (3.0% vs. 3.8%) and back squat exercises (4.3% and 3.1%). Larger fiber cross-sectional areas with more type II fiber characteristics are commonly found in males, whereas females have smaller fibers with more type I fiber characteristics [12,54]. At higher absolute isokinetic velocities, males produce more repetitions to fatigue than females. Conversely, females need less time to recover than males after moderate and fast isokinetic exercise [55]. However, sex differences in muscle fatigue under dynamic contractions are task-specific [56], and our study supports this notion since females reported a greater number of repetitions than males in both exercises (*p* < 0.05), despite producing a lower mean velocity and power output. Nevertheless, despite the sex differences in muscular endurance performance, CAF produced a similar ergogenic effect on this task, improving the number of repetitions, mean velocity and power production. Altogether, these results indirectly affirm that the mechanism of action of CAF can be mainly attributed to a central rather than local mechanism, probably due to greater motor unit activation [20]. This is further emphasized by the fact that if local mechanisms of CAF were responsible, sex differences in muscular endurance performance should be found due to the differences in muscle fiber distribution between males and females [12,54].

The major limitation of the present study was the impossibility of measuring plasma levels of caffeine and the CYP1A2 polymorphism. This measurement would have provided valuable information regarding sex differences in this supplement’s absorption and metabolization effect. Nonetheless, despite these limitations, this study provides data about the ergogenic effect of caffeine on muscular strength, power, and endurance performance in 38 female participants, a population largely underrepresented in the literature. Besides this, the present study provided direct evidence about the sex differences in the acute effects of caffeine on muscular strength, power, and endurance performance.

## 5. Conclusions

Low doses of CAF (3 mg/kg) have a similar ergogenic effect on muscular strength, power and endurance performance in upper- and lower-body exercises between male and female resistance-trained participants. Moreover, the ergogenic effect of CAF was more pronounced in mean and peak velocity and power output at moderate–high loads (50–90% 1RM) in the lower body (back squat exercise) irrespective of sex. Further studies are required to explore potential sex differences in muscular strength and power in upper-body exercises. This finding supports the use of CAF by sports nutritionists and dietitians to enhance muscular strength, power, or endurance performance in both male and female athletes, particularly in those actions that require the mobilization of moderate-to-high loads and involve larger muscle size groups.

## Figures and Tables

**Table 1:** Body composition, dietary and physical activity habits in each experimental group.

	CAF (N = 76)		PLA (N = 76)		ANOVA Effects		
	Females (N = 38)	Males (N = 38)	Females (N = 38)	Males (N = 38)	Sex (Partial η^2^)	Supplement (Partial η^2^)	Sex × Supplement (Partial η^2^)
	*Mean ± SD*	*Mean ± SD*	*Mean ± SD*	*Mean ± SD*			
***Body composition***							
Body mass (kg)	59.9 ± 7.1	78.5 ± 11.7	60.0 ± 7.2	78.2 ± 11.9	**<0.001 (0.634)**	0.564 (0.009)	0.955 (0.003)
Fat mass (kg)	12.6 ± 3.4	9.9 ± 5.1	12.7 ± 3.2	9.8 ± 5.2	**0.004 (0.213)**	0.882 (0.001)	0.257 (0.035)
Fat-free mass (kg)	47.3 ± 5.5	68.6 ± 7.9	47.3 ± 5.7	68.3 ± 7.9	**<0.001 (0.815)**	0.991 (<0.001)	0.758 (0.003)
***Dietary habits***							
Energy intake (kcal)	1270 ± 336	1972 ± 847	1264 ± 354	1959 ± 846	**<0.001 (0.436)**	0.415 (0.019)	0.929 (0.008)
Protein (g/kg)	1.30 ± 0.50	1.59 ± 0.89	1.28 ± 0.50	1.56 ± 0.85	0.186 (0.048)	0.306 (0.047)	0.560 (0.010)
Carbohydrate (g/kg)	2.28 ± 0.78	2.49 ± 1.50	2.27 ± 0.86	2.39 ± 1.28	0.761 (0.003)	0.538 (0.011)	0.818 (0.054)
Fat (g/kg)	0.82 ± 0.33	1.24 ± 0.91	0.84 ± 0.39	1.25 ± 0.86	0.290 (0.126)	0.989 (0.017)	0.833 (0.045)
***Physical Activity habits***							
METs-min/week	5027 ± 757	6048 ± 899	4906 ± 557	6725 ± 924	0.916 (<0.001)	0.775 (0.003)	0.478 (0.018)
Sedentary time (h/day)	7.40 ± 5.57	7.95 ± 7.78	7.38 ± 5.91	8.20 ± 6.42	0.379 (0.023)	0.346 (0.039)	0.349 (0.026)

Data are provided as mean ± standard deviation. Abbreviations: MET, metabolic equivalent of task; CAF, caffeine; PLA, placebo.

## Data Availability

The original contributions presented in the study are included in the article, further inquiries can be directed to the corresponding author.
